# Early Vasopressor Initiation Before Complete Fluid Resuscitation in Adults With Septic Shock: A Systematic Review

**DOI:** 10.7759/cureus.116968

**Published:** 2026-09-26

**Authors:** Ahmed Abdalla Mohammed Hamadtalla, Islam Mustafa Hamid Mohammed, Muhammad Abbas, Yusuf Elmi, Ala Ibrahim Elhashmi Elhbishi, Nahid Elamin Ahmed Elgailani, Omima Mohamed

**Affiliations:** 1 Intensive Care Unit, University Hospital Sharjah (UHS), Sharjah, ARE; 2 Acute Medicine, Sandwell and West Birmingham NHS Trust, Birmingham, GBR; 3 Intensive Care Unit/Internal Medicine, Mediclinic City Hospital, Dubai, ARE; 4 Emergency Medicine, South Warwickshire University NHS Foundation Trust, Warwick Hospital, Warwick, GBR; 5 Clinical Microbiology, Princess Royal University Hospital, Orpington, GBR; 6 General Practice, Omdurman Islamic University, Khartoum, SDN; 7 General Medicine, Bon Secours Hospital, Limerick, IRL

**Keywords:** fluid resuscitation, norepinephrine, restrictive fluid strategy, sepsis, septic shock, systematic review, timing of therapy, vasopressors

## Abstract

Guidelines suggest at least 30 mL/kg of crystalloid within three hours in septic shock but do not specify when vasopressors should begin in relation to that bolus. Hence, we systematically reviewed contemporary evidence on initiating vasopressors before fluid resuscitation is complete. For this review, early initiation was defined as commencement of a first-line vasopressor before the treating team or study protocol judged initial volume expansion complete, and every included study’s own time-zero anchor, threshold, and definition of completeness were extracted. PubMed, Scopus, Web of Science, and ClinicalTrials.gov were searched from 1 January 2021 to 18 August 2026, supplemented by citation searching. Reporting followed the Preferred Reporting Items for Systematic Reviews and Meta-Analyses 2020 guidelines, and synthesis followed Synthesis Without Meta-analysis. Eligible studies were randomised controlled trials (RCTs) and comparative observational studies in adults with septic shock or sepsis-induced hypotension, contrasting earlier with later vasopressor initiation relative to fluid loading. Risk of bias was assessed with Risk of Bias 2 and Risk of Bias in Non-randomized Studies - of Interventions, and certainty of evidence with the Grading of Recommendations Assessment, Development, and Evaluation. Heterogeneity in exposure definition precluded meta-analysis. In total, 11 studies (two multicentre RCTs and nine comparative observational studies; 18,362 adults; eight countries) were included. Thresholds for “early” spanned 25 minutes to three hours and were anchored variably to triage, shock recognition, or the first fluid bolus; completeness of fluid resuscitation was protocolised in only the two RCTs and left to clinician judgement in seven cohorts, with the pre-vasopressor fluid volume unmeasured in two. Both RCTs were neutral for their primary patient-centred endpoints (moderate certainty) despite achieving 1.1 to 2.1 L separation in 24-hour fluid volume (high certainty). Observational findings were discordant (very low certainty): four cohorts reported lower mortality with earlier initiation, one propensity-matched registry analysis reported higher 28-day mortality, and three found no association. Benefit was concentrated in patients meeting Sepsis-3 criteria, with high predicted vasopressor requirement, and with marked diastolic hypotension and a wide pulse pressure. No safety penalty was identified (moderate certainty). Initiating vasopressors before fluid resuscitation is complete is safe and reduces early fluid exposure and time to haemodynamic target, but does not improve survival when applied indiscriminately. The evidence supports the 2026 Surviving Sepsis Campaign position that concurrent vasopressor administration be reserved for case-by-case use in unstable shock, and future trials should enrol by vasoplegic phenotype rather than by time alone.

## Introduction and background

Sepsis is a life-threatening organ dysfunction caused by a dysregulated host response to infection, and septic shock is the subset in which underlying circulatory, cellular, and metabolic abnormalities are profound enough to substantially increase mortality [1]. Its epidemiological footprint is immense and has widened rather than narrowed. The most recent Global Burden of Disease (GBD) analysis estimated 166 million (95% uncertainty interval: 135-201) incident sepsis cases and 21.4 million (20.3-22.5) sepsis-related deaths worldwide in 2021, representing 31.5% of all global deaths, with the burden rising in 2020 and 2021 and reversing progress accrued since 1990 [2]. The preceding GBD 2017 analysis had already doubled earlier estimates, reporting 48.9 million incident cases and 11.0 million sepsis-related deaths, with the greatest burden concentrated in sub-Saharan Africa, Oceania, and South and East Asia [3]. Restricted to formally treated disease, a systematic review estimated approximately 48 cases of hospital-treated sepsis per 100,000 person-years and hospital mortality of 26.7%, rising to 41.9% among patients treated in intensive care units (ICUs) [4]. Sepsis is correspondingly the dominant reason for critical care occupancy: in the EPIC III point-prevalence study of 15,202 patients across 1,150 centres in 88 countries, 54% of ICU patients had suspected or proven infection, and ICU mortality among infected patients was more than double that of uninfected patients [5]. Within this population, septic shock carries the highest risk, with pooled short-term mortality in Europe, North America, and Australia between 2009 and 2019 of approximately 39% [6].

The cornerstone of initial haemodynamic management remains rapid intravenous crystalloid expansion. The 2026 Surviving Sepsis Campaign (SSC) guidelines retain the conditional recommendation to administer at least 30 mL/kg of intravenous crystalloid within the first three hours for sepsis-induced hypoperfusion or septic shock, and, for the first time, address the timing of vasopressor initiation as a discrete question, suggesting initial crystalloid bolus resuscitation followed by vasopressor support if hypotension persists, on very low certainty evidence, with a remark that immediate concurrent vasopressor administration may be warranted case by case in unstable shock [7]. The 2021 edition, which governed practice throughout the period covered by this review, was silent on the question [8]. Two definitions therefore require explicit statement before the literature can be interpreted. “Early vasopressor initiation” denotes commencement of a first-line vasopressor before initial volume expansion has been completed, that is, before, simultaneously with, or during the guideline-recommended bolus rather than after it has failed. “Complete fluid resuscitation” denotes the point at which volume expansion is judged finished, which may be operationalised as a fixed weight-based volume (≥30 mL/kg), a fixed protocolised volume, dynamic assessment of fluid responsiveness, lactate clearance, or unstructured clinician judgement. These two constructs are logically dependent on one another, yet neither is standardised across the literature, and much of the apparent disagreement between studies follows directly from that fact.

There are coherent physiological arguments on both sides. Septic shock is predominantly a vasoplegic state, and norepinephrine acts on venous capacitance vessels to convert unstressed to stressed blood volume, raising mean systemic filling pressure and augmenting venous return; in septic patients with life-threatening hypotension, early norepinephrine increases cardiac preload and cardiac output rather than depressing them [9,10]. Because arterial pressure responds faster and more predictably to a vasopressor than to a fluid bolus, initiating norepinephrine alongside, rather than after, volume expansion could shorten the cumulative duration of hypotension and reduce the total fluid required to reach a mean arterial pressure (MAP) of 65 mmHg, thereby avoiding the interstitial oedema, glycocalyx injury, and organ dysfunction that accompany a positive fluid balance. The countervailing concern is that vasoconstricting an underfilled circulation may raise arterial pressure while worsening regional and microcirculatory flow, and that earlier initiation lengthens total catecholamine exposure, which has itself been associated with worse outcomes in dose-dependent analyses [11].

Attempts to resolve this uncertainty empirically have produced a fragmented literature. A pilot randomised trial of restricted fluid resuscitation with earlier vasopressor commencement in emergency department (ED) patients with sepsis-associated hypotension demonstrated feasibility and safety but was not powered for mortality [12], while observational analyses have reached conflicting conclusions that plausibly reflect confounding by indication, as the sickest patients receive vasopressors soonest. Contemporary opinion has therefore shifted from asking whether early vasopressors are universally superior towards asking which patients should receive them first, with proposals for personalised, phenotype-guided initiation [13]. This shift, together with the recent completion of two large pragmatic randomised trials, the publication of several high-quality registry analyses, and the appearance of a dedicated guideline statement on vasopressor timing, makes a focused synthesis timely. The objective of this systematic review was to identify, appraise, and synthesise contemporary primary evidence comparing vasopressor initiation before or during fluid resuscitation with initiation after fluid resuscitation in adults with septic shock, with respect to mortality, resuscitation-related physiology, organ support, and safety, and to determine how the operational definitions of exposure and of resuscitation completeness influence the conclusions that can be drawn.

## Review

Methodology

Protocol and Reporting

This systematic review was designed, conducted, and reported in accordance with the Preferred Reporting Items for Systematic Reviews and Meta-Analyses (PRISMA) 2020 statement [14], and the narrative synthesis was reported in accordance with the Synthesis Without Meta-analysis (SWiM) guideline [15]. The review question, eligibility criteria, search strategy, data items, risk-of-bias approach, and certainty-of-evidence approach were specified a priori before formal screening began. Because the review used only published aggregate data, institutional ethics approval and informed consent were not required.

Operational Definitions of the Exposure and Resuscitation Completeness

Because the included literature uses incompatible terminology, two constructs were operationally defined in advance. Early vasopressor initiation was defined as commencement of a first-line vasopressor (predominantly norepinephrine) at any point before initial volume expansion was judged complete, whether that occurred before, simultaneously with, or during the initial crystalloid bolus. No single clock threshold was imposed, because no such threshold is used consistently in the field and imposing one would have excluded most of the relevant evidence. Instead, each study was required to make an explicit early-versus-late contrast, and for every study we extracted three items separately: the reference time point from which the interval was measured (time zero), the numerical threshold used to define “early,” and the volume of fluid actually delivered before vasopressor initiation.

Complete fluid resuscitation was defined as the point at which the treating clinician or the study protocol judged initial volume expansion to have been finished. Four possible operationalisations were prespecified: a fixed weight-based volume (≥30 mL/kg of crystalloid), a fixed protocolised volume administered before enrolment or randomisation, dynamic assessment of fluid responsiveness or lactate clearance, and unstructured clinician judgement. Each included study was classified against these four categories, and studies in which the pre-vasopressor fluid volume was neither protocolised nor measured were identified as such.

Eligibility Criteria

Eligibility was structured using the Population, Intervention/Exposure, Comparator, Outcomes, and Study design (PICOS) framework and is set out in Table 1. In brief, we included original peer-reviewed randomised controlled trials (RCTs) and comparative observational studies of adults (≥18 years) with septic shock or sepsis-induced hypotension in which one group received a first-line vasopressor earlier in the resuscitation sequence, as defined above, and a comparator group received it later, after fluid resuscitation had failed to restore perfusion pressure; at least one mortality or days-alive-and-out-of-hospital outcome was required. Reviews, meta-analyses, conference abstracts, editorials, letters, protocols, preprints, case reports, paediatric and animal studies, and studies of second-line adjunctive agents were excluded. Because an unrestricted scoping search identified more than 10 eligible studies, eligibility was restricted a priori to English-language articles published from 1 January 2021 onwards, confining the synthesis to practice contemporary with the Sepsis-3 definitions; landmark earlier studies are used as comparators in the Discussion rather than counted among the included studies.

**Table 1:** Population, Intervention/Exposure, Comparator, Outcomes, and Study design (PICOS) eligibility criteria.

Domain	Included	Excluded
Population	Adults ≥18 years with septic shock or sepsis-induced hypotension (Sepsis-3, Sepsis-2, or explicit study definition); any setting	Age <18 years; predominantly non-septic shock; inseparable mixed-shock cohorts; animal or in vitro studies
Intervention/Exposure	First-line vasopressor started before initial volume expansion was judged complete (before, with, or during the initial bolus), including restricted-fluid/vasopressor-first trial arms. Study must state its time-zero anchor and its “early” threshold	Timing of adjunctive second-line agents; comparisons of agent, dose, blood-pressure target, or route alone
Comparator	Later or conventional initiation after fluid resuscitation had failed to restore the target mean arterial pressure, including liberal-fluid/later-vasopressor trial arms	No contemporaneous comparator; single-arm or unadjusted historical-control studies
Outcomes	Mortality (in-hospital, 28-, 30-, or 90-day) or days alive and out of hospital; secondary: fluid volume, time to target mean arterial pressure, lactate clearance, organ-support-free days, length of stay, safety events	No clinical outcome reported; surrogate endpoints only; estimates not extractable
Study design	Randomised trials and comparative observational studies (cohort, registry, propensity-score or weighted analyses); English-language full text, 1 January 2021 to 18 August 2026	Reviews, meta-analyses, editorials, letters, conference abstracts, protocols, preprints, case reports, guidelines; publications before 2021; non-English

Information Sources and Search Strategy

PubMed, Scopus, Web of Science, and ClinicalTrials.gov were searched from 1 January 2021 to 18 August 2026. The complete search string used for each source, together with the filters applied and the date of the final search, is reproduced in Table 2 so that the search can be re-executed exactly. The strategy combined controlled vocabulary and free-text terms across four concept blocks, namely, population, exposure, timing, and fluid resuscitation, and no methodological filter was applied to retain observational designs. The reference lists of all included articles and of recent systematic reviews on vasopressor timing were hand-searched, and forward citation tracking was performed in Google Scholar. Full text was obtained for every report that passed title and abstract screening. Articles not available through institutional subscription were sought through interlibrary loan and, where necessary, by direct request to the corresponding author, so that no report was excluded on grounds of cost or access.

**Table 2:** Search strategy by source.

Source	Search string as executed	Limits applied
PubMed/MEDLINE	(“septic shock”[MeSH] OR “septic shock”[tiab] OR “sepsis”[MeSH] OR “sepsis-induced hypotension”[tiab]) AND (“vasoconstrictor agents”[MeSH] OR vasopressor*[tiab] OR norepinephrine[tiab] OR noradrenaline[tiab] OR vasoactive[tiab]) AND (timing[tiab] OR early[tiab] OR delayed[tiab] OR initiation[tiab] OR “time to”[tiab]) AND (“fluid therapy”[MeSH] OR “fluid resuscitation”[tiab] OR crystalloid*[tiab] OR “restrictive fluid”[tiab] OR “fluid bolus”[tiab])	2021/01/01 to 2026/08/18; English; humans; adults 19+ years. No publication type or study design filter applied
Scopus	TITLE-ABS-KEY((“septic shock” OR “sepsis-induced hypotension” OR sepsis) AND (vasopressor* OR norepinephrine OR noradrenaline OR vasoactive) AND (timing OR early OR delayed OR initiation OR “time to”) AND (“fluid therapy” OR “fluid resuscitation” OR crystalloid* OR “restrictive fluid” OR “fluid bolus”))	PUBYEAR > 2020; LANGUAGE English; document type Article
Web of Science Core Collection	TS=((“septic shock” OR “sepsis-induced hypotension” OR sepsis) AND (vasopressor* OR norepinephrine OR noradrenaline OR vasoactive) AND (timing OR early OR delayed OR initiation OR “time to”) OR (“fluid resuscitation” OR crystalloid* OR “restrictive fluid” OR “fluid bolus”))	Publication years 2021–2026; English; document type Article
ClinicalTrials.gov	Condition: “septic shock” OR “sepsis”; Intervention/treatment: vasopressor OR norepinephrine OR “fluid resuscitation”; Study status: completed with results posted or linked publication	First posted or results first posted 2021 to 2026. Registry records screened to identify completed trials with full-text publications
Citation searching	Backward hand-searching of the reference lists of all included articles and of systematic reviews on vasopressor timing, plus forward citation tracking of each included article in Google Scholar	Conducted after database screening; reported as a separate identification pathway

Study Selection

All records were exported to reference management software, and duplicates were removed electronically and then verified manually. Two reviewers independently screened titles and abstracts, and the full texts of all potentially eligible records were then independently assessed by the same two reviewers against the criteria in Table 1. Disagreements at either stage were resolved by discussion, with recourse to a third reviewer where consensus could not be reached. Reasons for exclusion at full-text stage were recorded and are summarised in the PRISMA flow diagram.

Data Extraction

A standardised, piloted data extraction form was used by two reviewers working independently, with discrepancies reconciled against the source article. Extracted items comprised first author, year, country and setting, study design and data source, enrolment period, sample size and group sizes, diagnostic criteria for sepsis and septic shock, the time-zero anchor, the numerical threshold defining early initiation, the way in which completeness of fluid resuscitation was operationalised, the fluid volume administered before and after vasopressor initiation, the comparator definition, the vasopressor agents used, the analytical approach to confounding, all reported mortality endpoints with effect estimates and precision, secondary and physiological outcomes, safety data, and prespecified subgroup analyses. Where an article reported both crude and adjusted estimates, the fully adjusted estimate was extracted as the primary result.

Risk-of-Bias Assessment

Risk of bias in randomised trials was assessed using the Cochrane Risk of Bias (RoB) 2 tool across the five domains of the randomisation process, deviations from intended interventions, missing outcome data, measurement of the outcome, and selection of the reported result, with an overall judgement of low risk, some concerns, or high risk [16]. Non-randomised studies were assessed with the Risk of Bias in Non-randomized Studies - of Interventions (ROBINS-I) tool across the seven domains of confounding, selection of participants, classification of interventions, deviations from intended interventions, missing data, measurement of outcomes, and selection of the reported result, with an overall judgement of low, moderate, serious, or critical risk of bias [17]. Because patients who are more severely ill receive vasopressors sooner, confounding by severity was prespecified as the dominant domain for every non-randomised study; studies were credited only where propensity-score matching, inverse probability of treatment weighting, or comprehensive multivariable adjustment incorporating a validated severity score had been applied, and were downgraded where baseline severity differed materially between groups after adjustment. Two reviewers assessed each study independently and resolved disagreements by consensus; judgements are displayed as traffic-light plots, and their influence on the conclusions is analysed explicitly in the Discussion.

Certainty of Evidence

Certainty of evidence was rated for each outcome domain using the Grading of Recommendations, Assessment, Development and Evaluations (GRADE) approach [18]. Randomised evidence began at high certainty and non-randomised evidence at low certainty, and each body of evidence was then considered for downgrading on risk of bias, inconsistency, indirectness, imprecision, and publication bias, and for upgrading on large effect size. Randomised and non-randomised evidence for mortality was rated separately rather than pooled, because the two bodies of evidence point in different directions and averaging them would obscure the reason.

Data Synthesis

A quantitative meta-analysis was not performed, and this decision was prespecified rather than post hoc. The included studies differ in the anchoring of time zero (ED triage or arrival, recognition of sepsis or septic shock, the first documented hypotensive episode, the first resuscitative fluid bolus, or prehospital contact), in the threshold used to define early initiation (25 minutes to three hours, a more than sevenfold range), in whether completeness of fluid resuscitation was protocolised, measured, or neither, in the comparator (a protocolised later-vasopressor strategy versus usual care), and in the timing and definition of the mortality endpoint (in-hospital, 28-day, 30-day, or 90-day death, and days alive and out of hospital). A pooled estimate across exposures that are not the same exposure would be statistically precise but clinically uninterpretable, and no amount of statistical heterogeneity assessment can repair a definitional mismatch of this kind. Following SWiM guidance [15], studies were therefore grouped by outcome domain, i.e., mortality, fluid exposure and haemodynamic response, organ support and length of stay, safety, and effect modification, and, within each domain, by study design, with randomised evidence presented before non-randomised evidence. Vote counting based statistical significance was avoided; instead, effect estimates are reported with the direction, magnitude, and precision given in the source article, and the standardised metric for synthesis was the adjusted effect estimate for mortality. Because no meta-analysis was undertaken, heterogeneity statistics such as I² and funnel plot or Egger tests for small-study effects were not calculable, and their absence is acknowledged as a limitation.

Results

Study Selection

The database and register searches yielded 185 records, of which 92 duplicates were removed before screening. Of the 93 records screened, 63 were excluded based on irrelevant titles and abstracts. In total, 30 reports were sought for retrieval, and all 30 were obtained, including one initially behind a paywall that was subsequently secured through interlibrary loan; of the 30 assessed for eligibility, 19 were excluded because the exposure or comparator did not address vasopressor timing relative to fluid resuscitation, and four were conference abstracts or review articles, leaving seven included studies from this source. Citation searching identified a further 14 records, all of which were retrieved and assessed; two were excluded as not published in English and eight for not meeting the inclusion criteria, contributing four further studies. Finally, 11 primary studies were included in the narrative synthesis (Figure 1).

**Figure 1:**
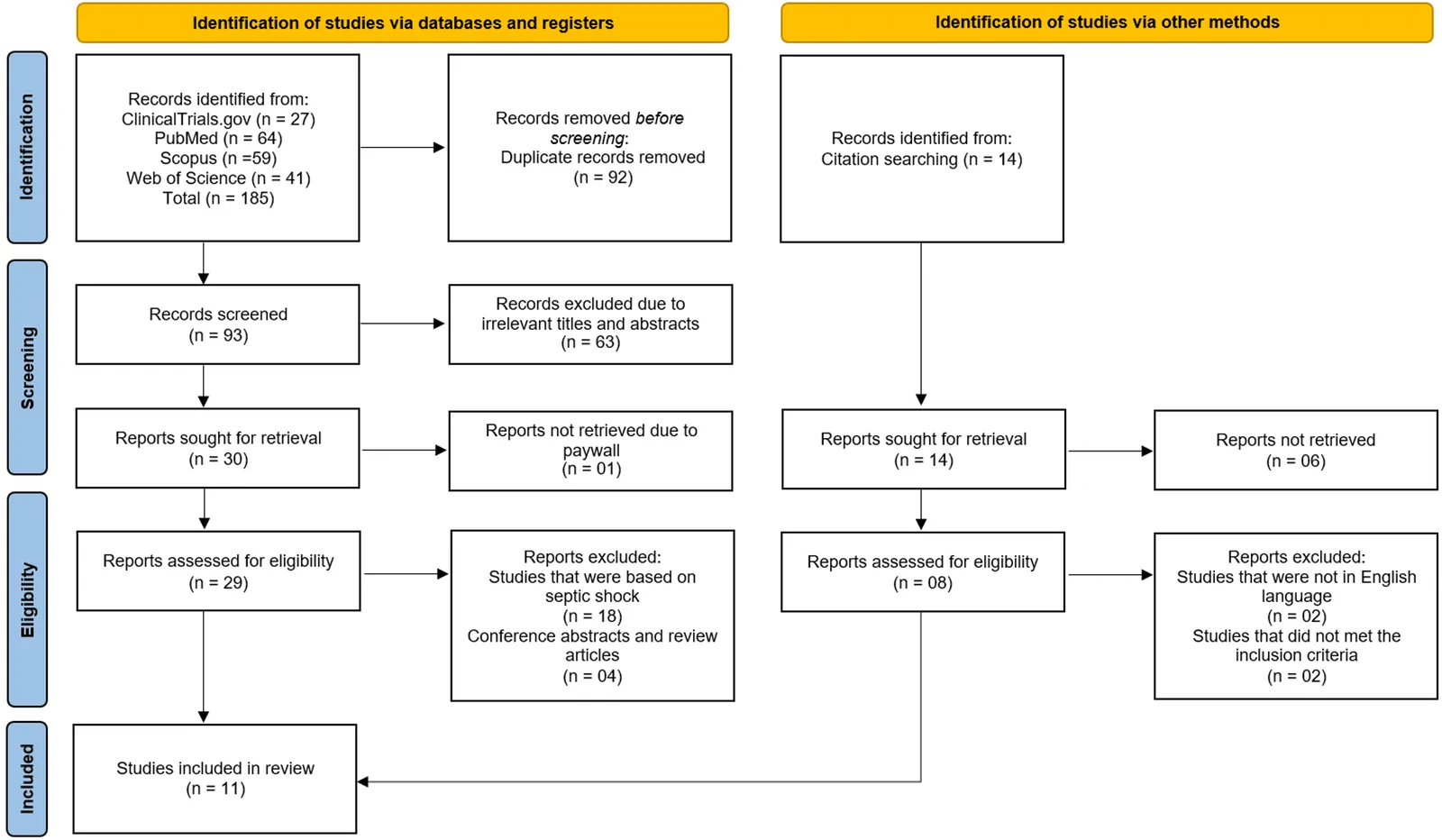
Preferred Reporting Items for Systematic Reviews and Meta-Analyses (PRISMA) 2020 flow diagram of study identification, screening, eligibility assessment, and inclusion

Characteristics of the Included Studies

The characteristics of the 11 included studies are summarised in Table 3. Two were multicentre RCTs, and nine were comparative observational studies, of which three used propensity-score matching or inverse probability of treatment weighting. Collectively, the studies enrolled 18,362 adults across eight countries, with individual sample sizes ranging from 97 to 4,699. In chronological order of publication, the included studies were a multicentre emergency department sub-study from Australia and New Zealand [19], a propensity-score analysis of the Medical Information Mart for Intensive Care (MIMIC)-IV database from the United States [20], a propensity-matched analysis of the Korean Sepsis Alliance registry [21], a prehospital cohort study from France [22], a multicentre Japanese ICU cohort [23], the Crystalloid Liberal or Vasopressors Early Resuscitation in Sepsis (CLOVERS) randomised trial from the United States [24], a single-centre prospective cohort from China [25], a statewide retrospective cohort from the United States [26], a registry analysis from the Korean Shock Society [27], a phenotype-stratified analysis of the Korean Sepsis Alliance registry [28], and the Australasian Resuscitation in Sepsis Evaluation: Fluid or Vasopressors in Emergency Department Sepsis (ARISE FLUIDS) randomised trial conducted in Australia, New Zealand, and Ireland [29].

**Table 3:** Characteristics of the 11 included studies. MIMIC-IV = Medical Information Mart for Intensive Care-IV; ICU = intensive care unit; HR = hazard ratio; IPTW = inverse probability of treatment weighting; RCT = randomised controlled trial; MAP = mean arterial pressure; OR = odds ratio; ED = emergency department; BP = blood pressure; aOR = adjusted odds ratio

Study	Country	Design	N	Definition of early initiation	Key finding
Kusakabe et al., 2021 [19]	Australia, New Zealand	Multicentre ED observational sub-study (70 sites)	177	≤2 hours from sepsis recognition or triage	Less fluid before vasopressor start (2.0 vs. 2.85 L); no difference in mortality or organ support
Xu et al., 2022 [20]	USA	Propensity-matched cohort (MIMIC-IV)	4,253	≤3 hours from shock onset	Lower 28-day mortality (30.0% vs. 37.8%); shorter ICU stay and ventilation
Yeo et al., 2022 [21]	South Korea	Prospective registry, 1:1 propensity-matched (16 sites)	415	≤1 hour from first fluid bolus	Higher 28-day mortality (47.7% vs. 33.6%); HR 1.83 (1.26–2.65)
Jouffroy et al., 2022 [22]	France	Prehospital cohort, propensity score and IPTW	478	Norepinephrine before hospital arrival	Lower 30-day mortality; adjusted HR 0.42 (0.25–0.70)
Abe et al., 2023 [23]	Japan	Multicentre ICU cohort, IPTW (17 sites)	97	≤1 hour from sepsis recognition	No mortality difference (32.8% vs. 26.7%); lower cumulative fluid trajectory
CLOVERS, 2023 [24]	USA	Multicentre open-label RCT (60 sites)	1,563	Restrictive fluid, vasopressor-first strategy for 24 hours	No difference in death before discharge home by day 90 (14.0% vs. 14.9%); 24-hour fluid −2,134 mL
Li et al., 2025 [25]	China	Single-centre prospective cohort	120	≤1 hour from shock onset	Lower 28-day mortality (28.6% vs. 47.4%); faster MAP target and higher lactate clearance
Black et al., 2025 [26]	USA	Statewide retrospective cohort (OneFlorida)	4,699	Shorter interval from first hypotension (continuous)	No association with 90-day mortality (OR 1.01 per hour) or vasopressor-free days
Choi et al., 2026 [27]	South Korea	Prospective registry analysis (21 EDs)	4,456	≤1 hour from ED arrival	Lower 28-day mortality in Sepsis-3 shock only; aOR 0.54 (0.35–0.81) for <1 h vs >6 h
Ko et al., 2026 [28]	South Korea	Prospective registry analysis, phenotype-stratified	1,104	≤25 minutes from ED triage	Lower mortality only if diastolic BP <50 mmHg; aOR 0.48 (0.25–0.90)
ARISE FLUIDS, 2026 [29]	Australia, New Zealand, Ireland	Multicentre open-label RCT (47 sites)	1,000	Restricted fluid with early vasopressors for 6–24 hours	No difference in days alive and out of hospital at day 90 (76 vs. 76); 24-hour fluid −1,108 mL

Definitions of Early Initiation and Complete Fluid Resuscitation

How each study operationalised the exposure and the endpoint of fluid resuscitation is set out in Table 4, and the heterogeneity is substantial enough to govern the interpretation of everything that follows. Time zero was anchored to ED triage or arrival in two studies [27,28], to recognition of sepsis or septic shock in three [20,23,25], to the first documented hypotensive episode in one [26], to the first resuscitative fluid bolus in one [21], and to prehospital contact with a mobile ICU in one [22]; one cohort used sepsis recognition and triage as alternative anchors [19], and the two randomised trials defined the exposure as allocation to a resuscitation strategy rather than to a fixed clock time [24,29]. Thresholds for “early” ranged from 25 minutes [28] through one hour [21,23,25,27] and two hours [19] to three hours [20].

**Table 4:** How each study defined early vasopressor initiation and the endpoint of fluid resuscitation. ED = emergency department; ICU = intensive care unit; MAP = mean arterial pressure

Study	Time zero	“Early” threshold	Fluid given before vasopressor start	How completeness of fluid resuscitation was operationalised
Kusakabe et al., 2021 [19]	Sepsis recognition or ED triage (both analysed)	2 hours	2.0 L (early) vs. 2.85 L (late); measured	Unstructured clinician judgement; no protocolised threshold
Xu et al., 2022 [20]	Septic shock onset	3 hours	Measured; concurrent with fluid resuscitation	Unstructured clinician judgement
Yeo et al., 2022 [21]	First resuscitative fluid bolus	1 hour	6-hour bolus 33.2 vs. 35.9 mL/kg; measured	Fixed weight-based volume; approximately guideline-concordant (≈30 mL/kg) in both arms
Jouffroy et al., 2022 [22]	Prehospital contact with mobile ICU	Before hospital arrival	Not reported	Clinician judgement within a prehospital protocol (fluid, then norepinephrine if MAP <65 mmHg)
Abe et al., 2023 [23]	Sepsis recognition	1 hour	Cumulative volume trajectory measured; lower in early group	Unstructured clinician judgement
CLOVERS, 2023 [24]	Randomisation	Strategy-based (24 hours)	1–3 L before randomisation; protocolised	Fixed protocolised volume before randomisation, then algorithm-driven
Li et al., 2025 [25]	Septic shock onset	1 hour	24-hour volume measured; lower in early group	Unstructured clinician judgement
Black et al., 2025 [26]	First documented hypotensive episode	Continuous (0–24 hours)	Volume at 6 and 12 hours measured and modelled as covariate	Unstructured clinician judgement
Choi et al., 2026 [27]	ED arrival	1 hour	Not available	Not defined; pre-vasopressor volume unavailable (stated limitation)
Ko et al., 2026 [28]	ED triage	25 minutes	Not available	Not defined; 3-hour bundle completion used only as a surrogate for resuscitation intensity
ARISE FLUIDS, 2026 [29]	Randomisation	Strategy-based (6–24 hours)	≈1–2 L before randomisation; protocolised	Fixed protocolised volume before randomisation, then algorithm-driven

Completeness of fluid resuscitation was defined even less consistently. Only the two randomised trials protocolised it, each randomising after a fixed initial volume of approximately 1 to 3 L had been given [24,29]. One cohort anchored the exposure directly to the first resuscitative fluid load and documented that both groups received an approximately guideline-concordant six-hour bolus volume of 33.2 versus 35.9 mL/kg, making it the only observational study in which the 30 mL/kg construct was verifiably met in both arms [21]. In five further cohorts, completeness rested on unstructured clinician judgement, although the fluid volume actually delivered was measured and reported [19,20,23,25,26]. In the two largest Korean registry analyses, the volume of fluid administered before vasopressor initiation was not available at all; one used completion of the three-hour sepsis bundle as a surrogate for resuscitation intensity [28], and the other reported the absence of these data as an explicit limitation [27]. No included study used dynamic assessment of fluid responsiveness or lactate clearance to define the endpoint of volume expansion. Most studies used norepinephrine as the index agent; one Australasian cohort reported that only a minority received norepinephrine as a single agent, with metaraminol frequently used first [19].

Mortality: Randomised Evidence

Effect estimates for all included studies are summarised in Table 5. Neither randomised trial demonstrated a survival benefit from prioritising vasopressors over fluid. In CLOVERS, 1,563 patients with sepsis-induced hypotension refractory to 1 to 3 L of intravenous fluid were randomised at 60 United States centres to a restrictive fluid strategy prioritising vasopressors (n = 782) or a liberal fluid strategy prioritising fluid before vasopressors (n = 781) for 24 hours [24]. The intervention achieved clear separation, with a difference in median 24-hour fluid volume of -2,134 mL (95% confidence interval (CI) = -2,318 to -1,949) and earlier, more prevalent, and longer vasopressor use in the restrictive group, yet death before discharge home by day 90 occurred in 109 (14.0%) patients versus 116 (14.9%) patients, an estimated difference of -0.9 percentage points (95% CI = -4.4 to 2.6; p = 0.61), and the trial was stopped early for futility [24]. ARISE FLUIDS randomised 1,000 adults presenting to emergency departments at 47 hospitals in Australia, New Zealand, and Ireland to restricted fluid with early vasopressors or higher-volume fluid with later vasopressors for at least six and up to 24 hours, with 963 patients in the intention-to-treat population [29]. The vasopressor group again received less fluid (median difference = -1,108 mL, 95% CI = -1,395 to -850) and were 18.9 percentage points more likely to receive a vasopressor (95% CI = 13.3 to 24.5), but the median number of days alive and out of hospital at day 90 was 76 in both groups (difference = 0.0 days, 95% CI = -2.7 to 2.7; p = 1.00) [29].

**Table 5:** Mortality and resuscitation outcomes across the included studies. ICU = intensive care unit; HR = hazard ratio; SOFA = Sequential Organ Failure Assessment; aHR = adjusted hazard ratio; aOR = adjusted odds ratio; IPTW = inverse probability of treatment weighting; SAE = serious adverse event; MAP = mean arterial pressure; BP = blood pressure

Study	Mortality effect estimate	Fluid and haemodynamic findings
Kusakabe et al., 2021 [19]	In-hospital mortality: no difference	Less fluid before vasopressor (2.0 vs. 2.85 L; p < 0.001); no difference in organ support
Xu et al., 2022 [20]	28-day mortality 30.0% vs. 37.8%; restricted mean survival 21.89 vs. 20.37 days	Shorter norepinephrine and ventilation duration; ICU stay 4.9 vs. 7.2 days
Yeo et al., 2022 [21]	28-day mortality 47.7% vs. 33.6% (p = 0.013); HR 1.83 (1.26–2.65)	6-hour fluid similar (33.2 vs. 35.9 mL/kg); higher day-3 SOFA (9.2 vs. 7.7) and lactate (2.8 vs. 1.7 mmol/L)
Jouffroy et al., 2022 [22]	30-day mortality: aHR 0.42 (0.25–0.70); IPTW aOR 0.75 (0.70–0.79)	Fluid volume not reported; no increase in adverse effects
Abe et al., 2023 [23]	In-hospital mortality 32.8% vs 26.7% (p = 0.543); aOR 0.76 (0.17–3.29)	Lower cumulative infusion trajectory; early group more severe (SOFA 10 vs. 9)
CLOVERS, 2023 [24]	Day-90 death before discharge home 14.0% vs. 14.9%; −0.9 pp (−4.4 to 2.6); p = 0.61	24-h fluid difference −2,134 mL (−2,318 to −1,949); earlier, longer vasopressor use; SAEs similar
Li et al., 2025 [25]	28-day mortality 28.6% vs. 47.4% (p = 0.045); initiation >1 hour an independent risk factor	Lower 24-hour fluid (p = 0.030); MAP ≥65 mmHg in 1.0 vs. 1.5 h; 6-hour lactate clearance 40.7% vs. 14.9%
Black et al., 2025 [26]	90-day mortality OR 1.01 per hour (1.00–1.02); no association	No effect on vasopressor-free days; no excess acute kidney injury
Choi et al., 2026 [27]	Sepsis-3 shock: aOR 1.07 per hour of delay (1.02–1.13); aOR 0.54 (0.35–0.81) for <1 hour vs. >6 hours; no association in non-Sepsis-3 group	Early group markedly more severe (lactate 5.2 vs. 3.8 mmol/L; SOFA 8.0 vs. 6.2)
Ko et al., 2026 [28]	Low diastolic BP: aOR 0.48 (0.25–0.90); preserved BP: aOR 0.93 (0.68–1.26); P for interaction 0.036	Greatest benefit with low diastolic BP and wide pulse pressure (aOR 0.60, 0.38–0.93)
ARISE FLUIDS, 2026 [29]	Days alive and out of hospital at day 90: 76 vs 76; 0.0 days (−2.7 to 2.7); p = 1.00	24-h fluid difference −1,108 mL (−1,395 to −850); vasopressor use +18.9 pp; no excess harm

Mortality: Non-randomised Evidence

The observational literature was markedly discordant. Four studies reported that earlier initiation was associated with lower mortality. In the MIMIC-IV propensity-score analysis of 4,253 patients, norepinephrine started within three hours of shock onset was associated with lower 28-day mortality (30.0% versus 37.8%) and longer restricted mean survival time (21.89 versus 20.37 days) irrespective of preload dependency [20]. In a French prehospital cohort of 478 patients managed by mobile ICU teams, norepinephrine titrated to a MAP above 65 mmHg before hospital arrival was associated with lower 30-day mortality after propensity adjustment (adjusted hazard ratio = 0.42, 95% CI = 0.25 to 0.70) and after inverse probability weighting (adjusted odds ratio = 0.75, 95% CI = 0.70 to 0.79) [22]. A single-centre Chinese prospective cohort of 120 ICU patients found that norepinephrine initiation within one hour of shock onset was associated with lower 28-day mortality (28.6% versus 47.4%; p = 0.045), and initiation beyond one hour was an independent risk factor for death on multivariable analysis [25]. In the Korean Shock Society registry analysis of 4,456 hypotensive ED patients, each hour of delay increased the odds of 28-day death among patients meeting Sepsis-3 criteria (adjusted odds ratio = 1.07 per hour, 95% CI = 1.02 to 1.13; p = 0.002), with an adjusted odds ratio of 0.54 (95% CI = 0.35 to 0.81) for initiation within one hour compared with beyond six hours [27].

In direct contrast, the propensity-matched analysis of the Korean Sepsis Alliance registry found that vasopressor initiation within one hour of the first resuscitative fluid load was associated with higher mortality [21]. Among 415 patients treated at 16 tertiary or university hospitals, 149 early patients were matched 1:1 to 149 late patients; the median interval from the initial fluid bolus to vasopressor was 0.3 versus 2.3 hours, and although the six-hour fluid bolus volume did not differ meaningfully, day-three Sequential Organ Failure Assessment (SOFA) score (9.2 versus 7.7) and day-three lactate (2.8 versus 1.7 mmol/L) were higher in the early group, and early initiation carried a hazard ratio for 28-day mortality of 1.83 (95% CI = 1.26 to 2.65) [21]. Three further studies were neutral. The Australasian ED sub-study of 177 patients found no difference in in-hospital mortality, duration of vasopressor infusion, or need for organ support [19]. The Japanese multicentre cohort of 97 ICU patients reported in-hospital mortality of 32.8% with initiation within one hour versus 26.7% with later initiation (p = 0.543; adjusted odds ratio = 0.76, 95% CI = 0.17 to 3.29), a comparison substantially limited by sample size and by markedly greater illness severity in the early group [23]. The largest neutral study, a statewide retrospective cohort of 4,699 patients in Florida, found that time from the first hypotensive episode to vasopressor initiation was not associated with 90-day mortality (odds ratio = 1.01 per hour, 95% CI = 1.00 to 1.02) and was not an independent predictor of vasopressor-free days, whereas age, mechanical ventilation, laboratory SOFA components, lactate, and liver disease were [26]. The ninth observational study reported an association entirely dependent on haemodynamic phenotype and is presented under effect modification below [28].

Fluid Exposure and Haemodynamic Response

In contrast to the discordant mortality signal, the effect of earlier vasopressor initiation on resuscitation physiology was consistent across designs, and every study that reported fluid volume found lower early fluid exposure with earlier vasopressor use. Both randomised trials achieved reductions of roughly 1.1 to 2.1 L over the first 24 hours [24,29]. In the Australasian cohort, patients in whom vasopressors were commenced early received substantially less intravenous fluid before vasopressor commencement (2.0 versus 2.85 L; p < 0.001) [19]. The Chinese prospective cohort reported a lower 24-hour infusion volume in the early group (p = 0.030) [25], and the Japanese cohort demonstrated a lower trajectory of cumulative infusion volume over time on mixed-effects modelling [23]. Haemodynamic response was likewise faster: the median time to achieve a MAP of at least 65 mmHg was shorter with early initiation (1.0 versus 1.5 hours; p = 0.010), and the six-hour lactate clearance rate was higher (40.7% versus 14.9%; p = 0.009) [25].

Organ Support and Length of Stay

Findings for organ support and length of stay were less consistent and were reported by fewer studies. The MIMIC-IV analysis found shorter durations of norepinephrine support and invasive mechanical ventilation and shorter ICU stay (4.9 versus 7.2 days) with early initiation [20]. The Chinese cohort found no difference in ICU or hospital length of stay [25], and the Australasian cohort found no difference in duration of vasopressor infusion or in requirement for organ support [19]. In the large Florida cohort, time to vasopressor initiation did not predict vasopressor-free days [26]. Neither randomised trial demonstrated a difference in patient-centred organ-support endpoints that would offset the neutral primary results [24,29].

Safety

No included study identified a safety penalty attributable to earlier vasopressor initiation. CLOVERS reported similar rates of serious adverse events between strategies, including low rates of complications related to peripheral vasopressor administration [24], and ARISE FLUIDS reported no excess of harm in the vasopressor group [29]. The propensity-matched Korean analysis, despite reporting higher mortality with very early initiation, did not attribute this to ischaemic complications [21], and the prehospital French cohort documented a mortality benefit without an increase in adverse effects [22]. Where renal outcomes were reported, no consistent increase in acute kidney injury or renal replacement therapy requirement was observed [20,26].

Effect Modification and Patient Selection

The most informative recent findings concern effect modification rather than average effect. The Korean Shock Society analysis showed that the association between earlier norepinephrine and lower 28-day mortality was confined to patients meeting Sepsis-3 septic shock criteria and to those at high predicted risk for vasopressor requirement based on diastolic shock index and lactate; in the non-Sepsis-3 group, no association was present (adjusted odds ratio = 0.96 per hour of delay, 95% CI = 0.88 to 1.04) [27]. Complementing this, the phenotype-stratified analysis of 1,104 ED patients in the Korean Sepsis Alliance registry found that norepinephrine initiated within 25 minutes of triage was associated with lower in-hospital mortality only among patients with a pre-vasopressor diastolic blood pressure below 50 mmHg (47.1% versus 62.5%; adjusted odds ratio = 0.48, 95% CI = 0.25 to 0.90; p = 0.023), with no benefit when diastolic pressure was preserved (adjusted odds ratio = 0.93, 95% CI = 0.68 to 1.26; p for interaction = 0.036) [28]. Within the low diastolic pressure group, benefit was concentrated in those with a wide pulse pressure of at least 25 mmHg (adjusted odds ratio = 0.60, 95% CI = 0.38 to 0.93), a profile consistent with vasoplegia and preserved stroke volume, and was absent when pulse pressure was narrow (p for interaction = 0.02) [28]. Several studies also documented that patients receiving vasopressors soonest were systematically more severely ill at baseline, with higher lactate, higher SOFA scores, and a higher diastolic shock index [23,27], quantifying the magnitude of confounding by severity in this literature.

Risk of Bias in the Included Studies

Risk-of-bias judgements for the two randomised trials are shown in Figure 2. CLOVERS was rated at low risk of bias in all five domains and low risk overall [24]. ARISE FLUIDS was rated at low risk for the randomisation process, missing outcome data, measurement of the outcome, and selection of the reported result, but raised some concerns in the domain of deviations from intended interventions because of the open-label delivery of the resuscitation strategy, giving an overall judgement of some concerns [29]. Neither judgement is severe enough to explain the neutral primary results, and both trials achieved and documented the intended separation in fluid and vasopressor exposure, so the randomised evidence can be taken at face value.

**Figure 2:**
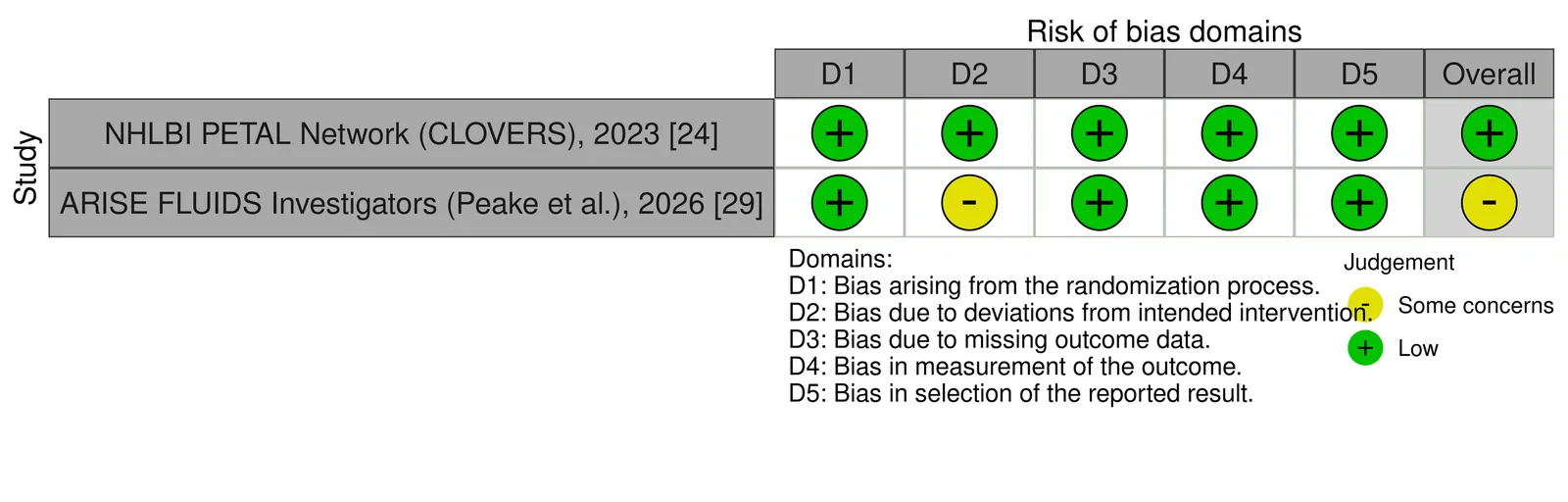
Risk-of-bias traffic-light plot for the two included randomised controlled trials (Cochrane Risk of Bias 2 tool).

Judgements for the nine non-randomised studies are shown in Figure 3. Six studies were rated at low risk of bias overall [20-22,25,27,28], reflecting prospectively collected registry or high-quality database sources combined with propensity-score matching, inverse probability of treatment weighting, or comprehensive multivariable adjustment including a validated severity score. One study was rated at moderate risk overall, with moderate concerns across the first five domains arising from reliance on administrative coding to identify sepsis and to time hypotension and vasopressor administration [26]. Two studies were rated at serious risk overall, in both cases driven by the confounding domain: the Australasian sub-study compared early and late groups without formal adjustment for baseline severity [19], and the Japanese cohort, although weighted by propensity score, was small and showed marked residual imbalance in illness severity between groups [23]. Measurement of outcomes and selection of the reported result were rated at low risk in all nine non-randomised studies.

**Figure 3:**
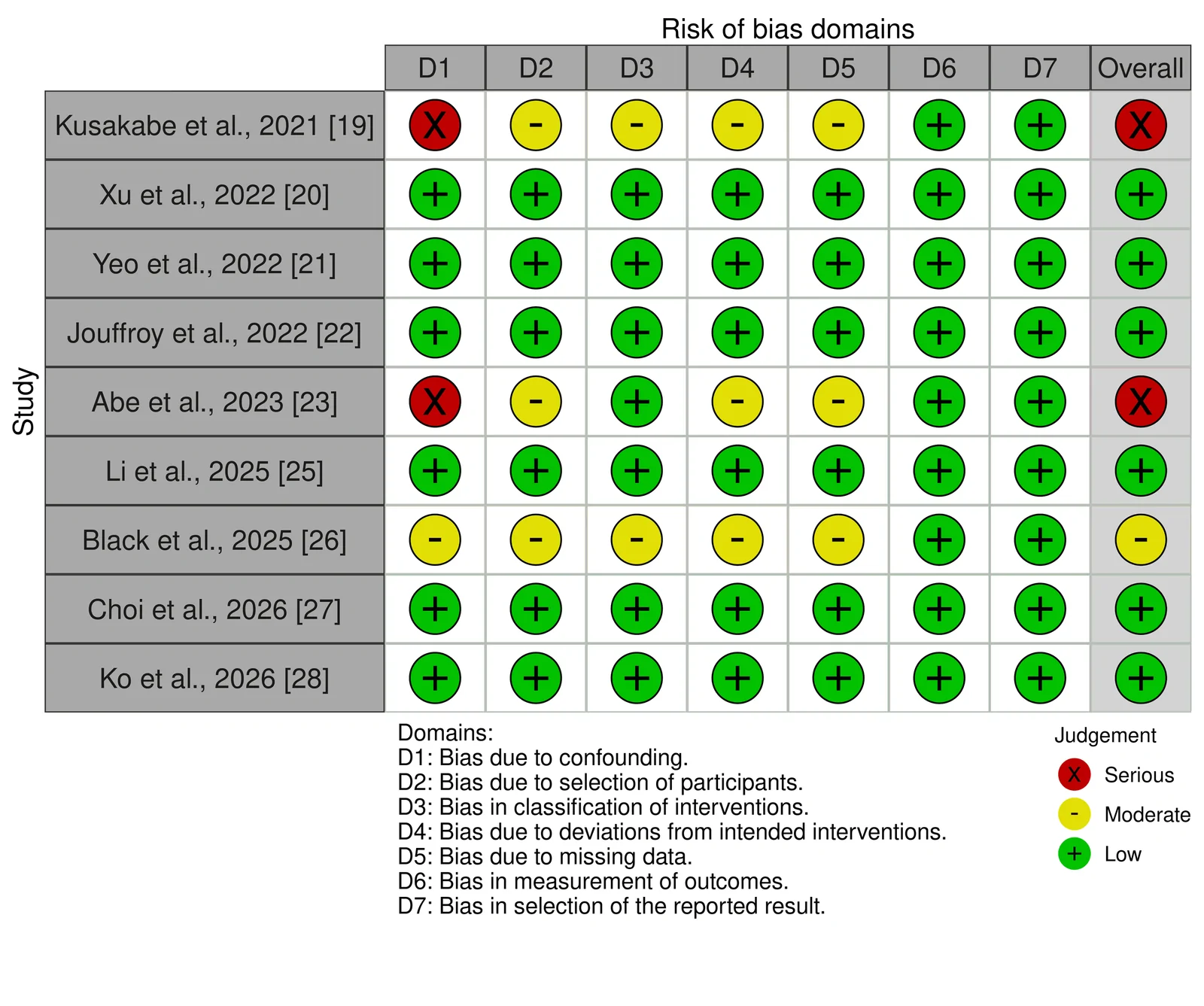
Risk-of-bias traffic-light plot for the nine included non-randomised studies (Risk of Bias in Non-randomized Studies - of Interventions-I tool).

Study quality maps directly onto the conclusions that can be drawn, and the mapping is informative rather than incidental. The two studies at serious risk of bias [19,23] are both neutral and small, so neither materially affects the overall picture in either direction. Of the two studies reporting the largest effects, one reported benefit [22], and one reported harm [21]; both were rated at low risk of bias on formal criteria, yet they reach opposite conclusions, which demonstrates that a favourable ROBINS-I rating certifies the adequacy of the analytical adjustment performed and not the absence of residual confounding by severity. The single moderate-risk study [26] is also the largest and most analytically neutral and found no association in either direction. Taken together, the non-randomised evidence therefore cannot arbitrate the question, and the synthesis rests principally on the randomised evidence, with the observational studies contributing chiefly to the identification of effect-modifying phenotypes.

Certainty of Evidence

GRADE ratings are presented in Table 6. Certainty was moderate that a protocolised vasopressor-prioritised strategy does not improve patient-centred survival outcomes in unselected patients, downgraded once for indirectness because both trials randomised only after an initial fluid bolus had been given. Certainty was high that earlier vasopressor initiation reduces 24-hour fluid volume, based on two concordant randomised trials with objectively measured exposure. Certainty was moderate that the strategy does not increase serious adverse events, downgraded once for imprecision because ischaemic and extravasation events were rare. By contrast, certainty for the non-randomised mortality evidence was very low, downgraded for inconsistency, because the estimates span a hazard ratio of 0.42 to 1.83 with no reconciling explanation other than differing exposure definitions and residual confounding, and for risk of bias. Certainty that earlier initiation shortens the time to the target MAP and improves six-hour lactate clearance was also very low, resting on a single small cohort [25].

**Table 6:** GRADE certainty of evidence by outcome domain. GRADE = Grading of Recommendations, Assessment, Development and Evaluations; RCT = randomised controlled trial; aHR = adjusted hazard ratio; MAP = mean arterial pressure

Outcome	Evidence base	Downgraded for	Certainty	Interpretation
Mortality/Days alive and out of hospital (randomised evidence)	2 RCTs; 2,563 patients [24,29]	Indirectness (−1): both trials randomised only after an initial 1–3 L bolus, so neither tested initiation before any fluid	Moderate	A protocolised vasopressor-prioritised strategy probably does not improve survival in unselected patients
Mortality (non-randomised evidence)	9 comparative cohorts; 15,799 patients [19-23,25-28]	Inconsistency (−1): estimates span aHR 0.42 to 1.83 with no reconciling explanation; risk of bias (−1): residual confounding by severity, exposure is itself a severity marker	Very low	The observational evidence cannot establish the direction of effect
Intravenous fluid volume at 24 hours	2 RCTs [24,29] with concordant cohort data [19,23,25]	Not downgraded; objective measurement, large and consistent effect	High	Earlier vasopressor initiation reduces 24-hour fluid volume by approximately 1.1 to 2.1 L
Time to target MAP and 6-hour lactate clearance	1 prospective cohort; 120 patients [25]	Imprecision (−1) and risk of bias (−1): single small single-centre cohort	Very low	Earlier initiation may shorten time to target MAP and improve lactate clearance
Safety (serious adverse events, ischaemia, acute kidney injury)	2 RCTs [24,29] with supporting cohort data [20,22,26]	Imprecision (−1): ischaemic and extravasation events were rare	Moderate	Earlier initiation probably does not increase serious adverse events

Discussion

Principal Findings

This systematic review of 11 contemporary primary studies encompassing 18,362 adults establishes a coherent picture from what has appeared to be an incoherent literature. The central finding is a dissociation between physiology and outcome. Initiating a vasopressor before fluid resuscitation is complete does what it is intended to do: it reliably reduces the volume of crystalloid administered in the first 24 hours, shortens the time to a target mean arterial pressure, and accelerates lactate clearance, and it does so without demonstrable ischaemic, renal, or arrhythmic harm [19,20,24,25,29]. What it does not do, when applied as a protocolised strategy to a broad population of patients with sepsis-induced hypotension, is improve survival or any patient-centred measure of recovery. Two adequately powered randomised trials, CLOVERS and ARISE FLUIDS, achieved substantial and verified separation in both fluid volume and vasopressor exposure and returned resoundingly neutral primary results [24,29]. The CIs around those estimates exclude large benefit and large harm alike. Taken together, these trials should end the debate about whether a vasopressor-first strategy is universally superior to a fluid-first strategy; it is not.

Comparison With Previous Literature

Our findings both extend and reframe the earlier evidence base. The Early Use of Norepinephrine in Septic Shock Resuscitation (CENSER) trial, a single-centre, double-blind, randomised trial of 310 patients in Thailand, remains the most methodologically rigorous demonstration of the physiological case: early low-dose norepinephrine markedly increased shock control at six hours (76.1% versus 48.4%; p < 0.001) and reduced cardiogenic pulmonary oedema (14.4% versus 27.7%; p = 0.004) and new-onset arrhythmia (11% versus 20%; p = 0.03), while 28-day mortality was numerically but not significantly lower (15.5% versus 21.9%; p = 0.15) [30]. CENSER was powered for a surrogate, and the pattern it established, i.e., unequivocal physiological benefit with an underpowered mortality signal, is precisely what the larger trials in the present review have now resolved in the direction of neutrality. Similarly, the propensity-score analysis by Ospina-Tascón and colleagues, in which 93 patients receiving vasopressors within one hour of, or before, the first resuscitative fluid load were matched to 93 receiving them later, reported a striking risk-adjusted reduction in day-28 mortality (hazard ratio = 0.31, 95% CI = 0.17 to 0.57) alongside dramatically lower fluid exposure (0 versus 1,500 mL at vasopressor start) [31]. Effect sizes of that magnitude from observational data are rarely reproduced in randomised trials, and the present synthesis illustrates why: the same design that generates the fluid-sparing signal cannot separate the effect of the intervention from the effect of the clinical judgement that prompted it.

The relationship between this review and the parallel literature on fluid restriction also deserves comment. The Conservative Versus Liberal Approach to Fluid Therapy of Septic Shock in Intensive Care (CLASSIC) trial randomised 1,554 ICU patients with septic shock who had already received at least 1 L of fluid to restrictive or standard fluid therapy and found identical 90-day mortality (42.3% versus 42.1%; adjusted absolute difference = 0.1 percentage points, 95% CI = -4.7 to 4.9; p = 0.96), despite a median difference in ICU fluid of roughly 2 L and with no excess of serious adverse events [32]. The pilot REFRESH trial reached compatible conclusions in the ED setting [12]. The consistent message across the fluid-restriction and vasopressor-timing literature is that, within the range of volumes and timings tested in modern practice, resuscitation strategy is far less determinative of survival than clinicians have assumed. That is a reassuring rather than a nihilistic conclusion: it means the fluid volume given and the moment a vasopressor is started can be individualised to physiology, comorbidity, and resource context without incurring a mortality penalty. Our synthesis is directly concordant with the position newly adopted by the 2026 SSC guidelines, which suggest initial crystalloid bolus resuscitation followed by vasopressor support if hypotension persists, on very low certainty evidence, while explicitly permitting immediate concurrent vasopressor administration on a case-by-case basis in patients with clinically unstable shock [7].

Confounding by Severity and the Influence of Study Quality

The single most important interpretive challenge in this literature is confounding by indication operating in both directions, and the risk-of-bias assessment is what allows that challenge to be quantified rather than merely asserted. Clinicians start vasopressors early in patients who look worst, which biases observational estimates towards harm, and they also start them early in units with mature sepsis pathways and rapid escalation cultures, which biases towards benefit. The included studies illustrate both directions vividly. In the Korean Shock Society cohort, patients receiving norepinephrine within one hour had substantially higher lactate (5.2 versus 3.8 mmol/L), higher SOFA scores (8.0 versus 6.2), and a higher proportion meeting Sepsis-3 criteria (77.9% versus 58.0%), and their crude mortality was correspondingly higher (31.6% versus 22.5%), yet the adjusted analysis reversed the direction of association entirely [27]. Conversely, the Korean Sepsis Alliance propensity-matched analysis by Yeo and colleagues, which matched on baseline covariates but analysed the interval from first fluid bolus to vasopressor, reported higher 28-day mortality with initiation within one hour [21]; the accompanying higher day-three SOFA and lactate values in the early group are as consistent with residual severity confounding as with iatrogenic harm.

That the largest and most methodologically neutral study, the 4,699-patient Florida cohort, found no association at all in either direction is instructive [26]. When the exposure is defined purely by elapsed time, and the population is unselected, the true average effect appears to be close to null, and observed associations largely track how well a given analytical approach has handled severity. This is why the formal risk-of-bias ratings must be read carefully rather than mechanically. Six of the nine non-randomised studies satisfied ROBINS-I criteria for low risk of bias, yet two of those six reach diametrically opposed conclusions [21,22]. A low ROBINS-I rating certifies that the investigators measured and adjusted for the confounders they could measure; it cannot certify that unmeasured severity has been eliminated, and in a field where the exposure is itself a marker of severity it almost certainly has not. We have therefore deliberately not allowed the favourable ratings of the observational studies to elevate their contribution to the synthesis. The GRADE assessment reflects this directly: the non-randomised mortality evidence is rated very low certainty despite predominantly low risk-of-bias judgements, and our principal conclusion rests on the two randomised trials, which are not susceptible to confounding by indication at all.

Defining the Exposure and the Endpoint of Resuscitation

A second explanation for trial neutrality lies in the comparator rather than the intervention, and it turns on how completeness of fluid resuscitation was defined. Both CLOVERS and ARISE FLUIDS randomised patients only after an initial fluid bolus of approximately 1 to 3 L had been administered [24,29]. Neither trial therefore tested vasopressor initiation before any fluid resuscitation; both tested how the remainder of the resuscitation should be conducted once a substantial volume had already been given. In CLOVERS, a substantial proportion of patients allocated to the restrictive arm never received a vasopressor at all, so the trial is in part a comparison of fluid volumes rather than of vasopressor timing. Our synthesis does not exclude these trials, because the clinical question posed in the review title is precisely about the sequence during and after the initial bolus, but the distinction is essential and is the basis on which we downgraded the randomised mortality evidence for indirectness. It also means the trials cannot exclude benefit from vasopressor initiation genuinely concurrent with the very first fluid, which is the strategy Ospina-Tascón and colleagues evaluated observationally [31] and which remains formally untested in a large randomised trial.

The methodological lesson generalises. The definitions of “early” used across the included studies span a more than sevenfold range, from 25 minutes to three hours, and time zero has been anchored to triage, to sepsis recognition, to shock recognition, to the first documented hypotension, and to the first fluid bolus. These are not interchangeable. A patient in whom norepinephrine is started 60 minutes after triage may have received it before any fluid or after 3 L, depending on the pathway. The definition of completeness is worse: only the two trials protocolised it, only one cohort verified that an approximately guideline-concordant volume had been delivered in both arms [21], five relied on unstructured clinician judgement, two could not measure pre-vasopressor fluid volume at all [27,28], and none used dynamic fluid responsiveness or lactate clearance. It follows that no study in this literature has actually tested the construct named in this review’s title with a measured, protocolised definition of resuscitation completeness, and the apparent disagreement between studies reflects measurement heterogeneity at least as much as biology. Standardised reporting of time zero, of the fluid volume delivered before vasopressor start, and of the haemodynamic phenotype at initiation should be a minimum expectation for future work in this area.

Clinical Implications

Several practical implications follow. First, because earlier vasopressor initiation reliably reduces fluid exposure without harm, it is a legitimate and defensible choice in patients in whom fluid tolerance is limited: those with heart failure, advanced chronic kidney disease, cirrhosis, or established respiratory failure. Second, because there is no survival benefit in unselected patients, there is no justification for a quality metric mandating vasopressor initiation within a fixed interval; such a metric would generate catecholamine exposure without benefit and would risk penalising appropriate clinical restraint. The finding that cumulative vasopressor dose is itself associated with mortality in dose-dependent analyses reinforces this caution [11], and the 2026 SSC guidelines make the same point in noting that conditional recommendations are poorly suited to rigid performance metrics [7]. Third, the practical barrier that historically justified delaying vasopressors, i.e., the need for central venous access, has largely dissolved: peripheral administration in early sepsis resuscitation is explicitly suggested by the 2026 SSC guidelines rather than delaying initiation until central access is secured [7], and was permitted by protocol in CLOVERS [24]. Fourth, the phenotype signals identified here are simple and immediately available at the bedside, as diastolic blood pressure, pulse pressure, and lactate require no specialist equipment and could be embedded in triage pathways at negligible cost [27,28].

The phenotype findings deserve emphasis because they reconcile the apparent conflict in the literature. Choi and colleagues showed that the association between earlier norepinephrine and survival was present only in patients meeting Sepsis-3 septic shock criteria and in those with a high predicted vasopressor requirement, and was entirely absent in less severe patients [27]. Ko and colleagues showed that the association was present only when the pre-vasopressor diastolic blood pressure was below 50 mmHg, and within that group was concentrated in patients with a wide pulse pressure, a combination that physiologically denotes profound vasoplegia with preserved stroke volume [28]. This is exactly the phenotype in which norepinephrine should be expected to help most: the pump is working, and the vasculature is failing, so restoring tone restores perfusion pressure within minutes, and the accompanying rise in diastolic pressure improves coronary perfusion. Conversely, in a patient with a narrow pulse pressure, a low-flow state predominates and vasoconstriction risks raising pressure while reducing flow, so preload optimisation or inotropy should take precedence. Framed this way, the neutral randomised trials and the positive phenotype-stratified cohorts are not in conflict; the trials averaged a heterogeneous treatment effect across populations in which the intervention should help, should be neutral, and might harm.

Knowledge Gaps and Future Research Directions

Important knowledge gaps remain. The most consequential is the absence of any randomised trial enrolling patients based on a vasoplegic phenotype rather than elapsed time or a fixed fluid threshold. Both CLOVERS and ARISE FLUIDS were pragmatic and deliberately unselective, which maximised generalisability at the cost of diluting a heterogeneous treatment effect; the logical next step is an enrichment design randomising patients with diastolic hypotension and preserved pulse pressure to immediate versus conventional vasopressor initiation, with days alive and free of organ support as the primary endpoint. Encouragingly, the 2026 SSC guidelines identify five geographically diverse randomised trials of vasopressor timing currently underway, whose results should substantially reduce the uncertainty documented here [7]. Second, no included study measured the microcirculation. The theoretical hazard of early vasopressors is that macrocirculatory pressure is restored at the expense of regional flow, yet none measured sublingual microcirculatory perfusion, splanchnic flow, or capillary refill dynamics, so the mechanism by which early initiation might harm an underfilled patient remains inferred rather than demonstrated. Third, no included study reported outcomes beyond 90 days, leaving long-term functional recovery, health-related quality of life, and post-sepsis morbidity unaddressed; ARISE FLUIDS has prespecified six- and 12-month disability outcomes whose publication will be informative. Fourth, all 11 studies were conducted in high-income or upper-middle-income settings with immediate access to vasopressor infusions and monitored beds, and the applicability of a vasopressor-first strategy in resource-limited environments, where fluid is cheap and infusion pumps and monitoring are scarce, is entirely unstudied, which is a gap of considerable global importance given that most sepsis deaths occur in such settings [2,3]. Finally, future studies should report the exposure using a standardised definition, ideally anchored to the onset of documented hypotension and accompanied by the measured pre-vasopressor fluid volume, so that the next synthesis can pool rather than narrate.

Limitations

This review has several limitations. First, quantitative pooling was not performed, for the prespecified reasons set out in the Methodology; consequently, no summary effect estimate and no assessment of statistical heterogeneity or small-study effects is provided, and the synthesis is necessarily narrative. Second, eligibility was restricted a priori to studies published from 1 January 2021 onwards. Although prespecified and confining the synthesis to practice contemporary with the Sepsis-3 definitions, this restriction excludes landmark earlier work including the CENSER and REFRESH randomised trials and the propensity-score analysis by Ospina-Tascón and colleagues; these are discussed in detail but do not contribute to the primary synthesis. Third, nine of the 11 included studies were non-randomised, and although most were formally rated at low risk of bias on account of prospective data collection and rigorous statistical adjustment, residual confounding by severity cannot be eliminated in any observational analysis in which the exposure is itself a marker of illness severity; these estimates are therefore treated as hypothesis-generating and were rated very low certainty on GRADE. Fourth, three of the included studies drew on Korean national sepsis registries, two of them from the same registry with partially overlapping enrolment periods; although the exposure definitions, analytical approaches, and eligible populations differed, duplication of contributing centres and of individual patients cannot be excluded, and a single healthcare system is therefore over-represented. Fifth, only English-language articles were eligible and two otherwise potentially relevant reports were excluded on this basis, raising the possibility of language bias; no report was excluded for reasons of access or cost. Sixth, the central construct of the review, i.e., vasopressor initiation before complete fluid resuscitation, could not be applied uniformly, because completeness of resuscitation was protocolised in only two studies and unmeasured in two others, so the synthesis necessarily describes a family of related exposures rather than a single one. Finally, two of the included reports were available only as online-ahead-of-print publications at the time of synthesis, so final pagination and any post-publication corrections were unavailable.

## Conclusions

Among adults with septic shock, initiating vasopressor therapy before fluid resuscitation is complete is safe and reproducibly achieves its intended physiological effects, reducing early crystalloid exposure, shortening the time to the target MAP, and improving early lactate clearance. It does not, however, improve survival or patient-centred recovery when applied as a uniform strategy to unselected patients, as demonstrated with moderate certainty by two adequately powered randomised trials with concordant neutral results. The discordance among observational studies is best explained by confounding by severity and by profound heterogeneity in how both “early” initiation and “complete” fluid resuscitation have been defined, and the certainty of that body of evidence is very low. The most credible signal in the contemporary evidence is that benefit is confined to a definable subgroup: patients with true Sepsis-3 septic shock, a high predicted vasopressor requirement, and a vasoplegic haemodynamic profile characterised by marked diastolic hypotension with a preserved or wide pulse pressure. Clinical practice should therefore follow a physiology-guided rather than protocolised sequence, consistent with the 2026 Surviving Sepsis Campaign position that concurrent vasopressor administration be reserved for case-by-case use in clinically unstable shock. Future randomised trials should enrol by phenotype rather than by the clock, should report time zero and the measured pre-vasopressor fluid volume in a standardised manner, and should extend follow-up beyond 90 days and into resource-limited settings.
